# Communication of Pulmonary Air Vesicles by a Direct Route with the Pulmonary Veins

**Published:** 1843-05

**Authors:** W. E. Horner

**Affiliations:** Prof. of Anatomy in the University of Pennsylvania


*Communication of Pulmonary Air Vesicles by a direct route with the Pulmonary Veins.* By W. E. Horner, M. D., Prof. of Anatomy in the University of Pennsylvania.—The following experiments go to determine this point.

Exp. I. In July last I lost a patient, James Roomy, ætat. 19, at the Philadelphia Hospital, who had been treated by me for calculus of the bladder, by lithotrity. On the examination of him after death finding the lungs in a state of perfect health, they were removed and taken to the University. I then fixed a pipe into the trachea and permitted a column of water to pass gently. The lungs filled up very completely, the air cells became distended with water, and somewhat to my surprise at the time, (for I had no such result in view, but merely to wash the lungs well for ulterior anatomical purposes,) the left side of the heart filled and the aorta began to discharge water from its cut branches very freely, in fact in a strong jet when compression was made so as to reduce the size of the stream in its exit.

No stream made its appearance from the right side of the heart, the water not showing any current in that direction, not even in drops, nor filling the pulmonary artery.

These observations were repeated at several trials of a separate kind on the same pair of lungs on the same occasion, and were renewed the next day. The result was announced at the time to Dr. Samuel Jackson, my colleague as we were in consultation on a patient.

Exp. II. In December last, a young athletic man lost both his legs from a railroad accident, followed by amputation. Having got his lungs with the heart attached, I renewed my experiments on the free communication of the air vesicles of the lungs with the pulmonary veins, and found the same results from a column of water gently let into the trachea—the left side of the heart readily filled, and the branches of the aorta spouted out water. The pulmonary artery and the right side of the heart did not fill, but a little water after a while returned by them, not however in any approximation to the quantity discharged by the aorta.

Exp. III. *Feb. 16th.* A Malay about thirty years of age, athletic and well formed, belonging to an East Indiaman, committed suicide about a fortnight ago. He stabbed himself in the abdomen so as to sever the colon and duodenum, and also opened the external carotid artery; by these several wounds he was well drained of blood. He was injected so as to retard putrefaction; the weather also has been highly favorable from its coldness, to the same end. The lungs today were in a state of perfect soundness, and of elegant normal texture and colour, so that I exhibited to the anatomical class the best specimen of them in a state of exact physical soundness with freedom from congestion of blood, that I have met with in thirty years of anatomical pursuits. In the evening I injected the left lung according to my plan, with tallow, having in view the connection of the air vesicles with one another.

On the 17th, I applied as in the two preceding experiments a column of water upon the lung of the right side. In a very little time I found the water returning by the pulmonary veins *pleno rivo*, and a very scanty show of it in the right pulmonary artery, not enough to discharge except by drops in a very slow manner. The experiment was renewed on several trials, and the results the same.

It may here be remarked that the return of the fluid by the pulmonary veins was much more rapid in the beginning than towards the end of these experiments, for when the lungs become infiltrated with the water the connection of the air vesicles and the pulmonary veins is not so free.

Exp. IV. *Sept.* 1842. On the lungs of a hog taken from a slaughter-house, the experiment done in the same way showed no communication either with pulmonary veins or arteries on the part of the air vesicles.

Exp. V. On the lungs of a sheep, resulted in failing to show also any communication with the pulmonary arteries and veins on the part of the air vesicles.

Exp. VI. On the lungs of a calf, also failed to prove the communication between the air vesicles and the pulmonary blood-vessels.

Among the venerable rites of that ancient people, the Hebrews, is a scrupulous regard to the perfect healthiness of the flesh that they eat, and also to the animal when slaughtered having the blood almost thoroughly evacuated. At the suggestion of my friend, the editor of this Journal, I obtained the lungs of animals prepared for market by one of their butchers, and the following results occurred in the lungs of the calf and sheep.

*Feb. 25th.* Exp. VII. On a calf. The lungs, upon the introduction of water into their air vesicles, began to return the water in a little time by the pulmonary veins and the pulmonary arteries. In keeping up the pressure of the column, it returned by a large free stream from both sets of vessels.

Exp. VIII. On a sheep. The lungs under the same regulated pressure of a column of water, returned it by the pulmonary arteries and veins also in a clear large stream.

In neither animal, however, did it return with equal freedom as in the human subject: though, in the lungs of the calf, the stream was sufficiently copious to wash back several large coagula of blood from the pulmonary artery.

The preceding experiments would go to prove the existence of a direct communication between the air vesicles and the pulmonary blood-vessels, especially the veins. A suggestion to the contrary, which may have some force, is, that the connection, as above established, is not by direct inosculation, but by infiltration: to which it may be replied, that in such case the injected fluid, by passing into the common connecting cellular substance, would constitute an intervesicular and interlobular dropsy, which would show itself by the water raising up the pleura in large vesications or bags—and by its forming large interlobular collections—also by the incapacity of the lungs to contract to the normal size in a short time, after the pressure of the water was withdrawn and the trachea left open. The lungs would at least remain for a time of a size nearly stationary on the suspension of the experiment, as in the carnification arising from the large effusions of blood into its substance in violent pneumonia. Now if prudence be observed in the experiment, none of these events occur, but the lungs collapse almost as readily as if they had been distended simply with air.

Exp. IX. On the fresh lungs of a large bullock. The residuary air of the pulmonary vesicles was, by the force of a column of water, driven from the lungs into the pulmonary blood-vessels, and the pulmonary artery was distended and inflated with the condensed air, so as to give it a tension and elasticity like that of a strongly inflated large intestine. With this state of things there was no emphysema of the lungs, which would follow inevitably without a direct communication between their air vesicles and the pulmonary vessels.

In the same lungs the current of water sent into them through the trachea, returned so freely by the pulmonary artery as to make a jet six or eight inches long, and of the size of the little finger; it also came out in a free jetting stream from the branches at the arch of the aorta. These jets could be increased, diminished or stopped without delay by the turning of a stop-cock.

Exp. X. On the lungs of a pig. I first of all inflated them by the pressure of a column of water acting on a reservoir of air. The air was found to pass readily to the heart by both pulmonary veins and arteries, but with especial freedom by the latter and distended them strongly. Having satisfied myself of this result, I then let in a column of water, which, as in the other experiments, returned freely by the pulmonary blood-vessels, being indicated by a bold stream from the pulmonary artery and aorta.

The abundance of the pulmonary capillaries, and their thinness and superficial position may be considered as additional arguments in favor of the conclusion drawn above, of direct inosculation with the air vesicles, but of course by pores which must be exceedingly fine. The lateral pressure of a column of water upon them would, without such inosculation, have the effect of water in the bladder upon the ends of the ureters, and would prevent itself from getting into them.

The foregoing experiments may serve to elucidate some of of the phenomena of respiration and of pulmonary hemorrhage.

The fact appears to be overlooked by pathologists generally, that the bright color of the blood in hæmoptysis, and the more superficial position and greater numbers of the pulmonary capillary veins, indicate that they are the true fountains of its blood instead of the arteries:—opinions which have for many years past been taught by me, on the ground of my minute injections of the lungs.

I may also state that with these experiments of the unquestionable transmission of fluids and of air into the pulmonary vessels from the air vesicles, we can now account for what every experienced anatomist has often observed, and will in every case see, to some extent: that there is always air in the pulmonary artery, the left side of the heart, and the aorta, after death, however recent the death may have been, and also account for the mistake of the ancients that the arteries conveyed air naturally, inasmuch as they were found filled with it after death, an error which has been indefectibly commemorated in the name of these tubes.

As leisure offers I propose to go on with the experiments on the above question of continuity between the blood-vessels and the vesicles of the lungs; but hope that in the mean time it may attract the inquiries of others more competent to settle the precise mode of this continuity.—*Amer. Journ. of the Med. Sci.*

